# An insight into lung cancer: A comprehensive review exploring anaplastic lymphoma kinase tyrosine kinase inhibitors and mechanisms of resistance

**DOI:** 10.17305/bjbms.2021.5859

**Published:** 2021-05-15

**Authors:** Adela Pătcaş, Ana Florica Chis, Claudia Florentina Militaru, Ioana Roxana Bordea, Ruxandra Rajnoveanu, Ovidiu Florin Coza, Reem Hanna, Tamaş Tiberiu, Doina Adina Todea

**Affiliations:** 1 Department of Medical Sciences, Pneumology, Faculty of Medicine, “Iuliu Hatieganu” University of Medicine and Pharmacy, Cluj Napoca, Romania; 2 Department of Pharmacology, Toxicology and Clinical Pharmacology, “Iuliu Hatieganu” University of Medicine and Pharmacy, Cluj-Napoca, Romania; 3 Medisprof Cancer Center, Cluj Napoca, Romania; 4 Department of Oral Rehabilitation, “Iuliu Hatieganu” University of Medicine and Pharmacy, Cluj-Napoca, Romania; 5 Department of Medical Oncology and Radiotherapy, “Iuliu Hatieganu” University of Medicine and Pharmacy, Cluj-Napoca, Romania; 6 Institute of Oncology “Prof. Dr. I. Chiricuta”, Cluj Napoca, Romania; 7 Department of Surgical Sciences and Integrated Diagnostics, Laser Therapy Centre, University of Genoa, Genoa, Italy; 8 Department of Oral Surgery, Dental Institute, King’s College Hospital NHS Foundation Trust, London, United Kingdom; 9 Department of Oral and Maxillofacial Surgery, “Iuliu Hatieganu” University of Medicine and Pharmacy Cluj-Napoca, Cluj Napoca, Romania

**Keywords:** Non-small cell lung cancer, anaplastic lymphoma kinase, anaplastic lymphoma kinase, tyrosine kinase inhibitors, resistance mechanisms

## Abstract

Implementation of precision medicine in lung cancer has benefited from intense research in the past years, developing subsequently an improved quality of life and increased overall survival of the patients. Targeted therapy has become one of the most important therapeutic innovations for the non-small cell lung cancer category with anaplastic lymphoma kinase (ALK) gene rearrangement. The aim of this review is to provide a thorough overview of the main molecules of ALK tyrosine kinase inhibitors (TKI) with their general and particular mechanisms of resistance, the main methods of ALK gene detection, each with advantages and limits, and the future perspectives currently under research which try to overcome the mechanisms of resistance. We have used two of the most reliable medical databases EMBASE and PubMed to properly select the latest and the most relevant articles for this topic. Encouraged by the promising results, the clinical practice was enriched by the approval of TKI molecules, three generations being developed, each one with more powerful agents than the previous ones. Unfortunately, the resistance to TKI eventually occurs and it may be induced by several mechanisms, either known or unknown. Crizotinib was the most intensely studied TKI, becoming the first molecule approved into clinical practice and although four other drugs have been broadly used (alectinib, ceritinib, brigatinib, and lorlatinib) it seems that even the most recently developed one remains imperfect due to the resistance mutations that developed. There are two types of resistance generally described for the entire class and for the particular drugs, but half of them remain unknown.

## INTRODUCTION

Lung cancer has gained a top place in the cancer-related incidence and mortality worldwide. Non-small cell lung cancer (NSCLC) which represents almost 80-85% of the patients is considered a heterogeneous disease due to the wide spectrum of molecular targets identified which have benefited from personalized therapy in recent years [1,2]. To better select the patients for this type of treatment, a proper method of anaplastic lymphoma kinase (ALK) gene detection must be used according to the diagnosis standards and the main options applied in clinical practice are fluorescence *in situ* hybridization (FISH), immunohistochemistry (IHC), reverse transcriptase-polymerase chain reaction (RT-PCR), next-generation sequencing (NGS), liquid biopsy, and new potential biomarkers such as circulating tumor cells (CTCs), cell-free DNA, and exosomes are being investigated [3]. The therapeutic development in this domain has led to the implementation of three generations of ALK tyrosine kinase inhibitors (TKI) with five molecules approved into clinical practice and two more molecules, such as entrectinib and ensartinib, are waiting for clinical approval [4]. The development of the ALK TKI in NSCLC patients, beginning with crizotinib, the first molecule approved and followed by second and third-generation inhibitors (ceritinib, alectinib, brigatinib, and lorlatinib) has provided an increased progression-free survival (PFS) and overall survival (OS) in comparison with chemotherapy [5,6]. Unfortunately, despite the initial benefit of ALK TKI, resistance mechanisms have been identified upon progression [7,8]. From the available data, we have discovered that almost half of the mechanisms of resistance remain unknown at this moment, but we have tried to present the recognized mutations and the future perspectives, even though they are under research. To properly identify the mechanisms, we have encompassed the main detection methods implemented into clinical practice or in preclinical data. Besides the particular mutations for each molecule, we have also described the factors which determine secondary mutations for the entire class [9,10]. Regarding the challenges of overcoming resistance, promising results are represented by the combination between ALK and epidermal growth factor receptor (EGFR) TKI, metformin, MYC (avian myelocytomatosis viral oncogene homolog) – inhibitors, human EGFR (HER) family, mammalian target of rapamycin (mTOR) pathway, anti-angiogenesis factors, etc. Nevertheless, the most awaited results are for the combination between ALK TKI and immunotherapy, which represents the most innovative therapeutic option for NSCLC patients [11-14].

## MATERIALS AND METHODS

**FIGURE 1:**
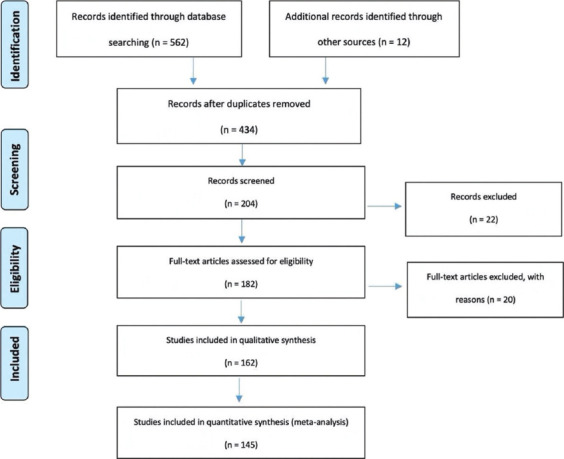
Description of the literature identification process (A Preferred Reporting Items for Systematic Review and Meta-analysis flowchart) [15].

In the present review, we have tried to encompass the most relevant and accurate available data from the literature regarding ALK TKI mechanisms of resistance in NSCLC patients. Our working hypothesis is based on describing the mechanisms of resistance for each molecule approved, presenting the advantages and disadvantages of the main detection methods currently used in clinical practice, and describing the future possibilities identified or still under research to provide therapeutic benefit. Our process of selection included two of the most reliable databases in the medical field, EMBASE, and PubMed, under specified criteria such as 10-years filter, English language, and Human species following A Preferred Reporting Items for Systematic Review and Meta-analysis statement as mentioned in Figure 1, but the final article selection remained subjective [15].

## RESULTS

### The role of ALK gene rearrangement in NSCLC

ALK gene has an incidence of 3-7% in patients with NSCLC and although more than 27 variants of ALK fusion proteins have been discovered, the most common partner is EML 4 (echinoderm microtubule-associated protein-like 4) [16,17]. The EML4-ALK fusion gene results from the paracentric inversion of chromosome 2 with at least 15 variants identified, variant 1 (v1) involving exon 13 being the most common, followed by the variant 3a/b (v3a/b) affecting exon 6 and variant 2 involving exon 20 [18,19]. This category of patients with ALK rearrangement has the following clinicopathological characteristics: Young age at diagnosis (a median of 50 years old), women gender, non-smokers/light smokers, histology of adenocarcinoma with particular morphologic patterns such as and cribriform and solid signet ring, expression of thyroid transcription factor 1, tendency to metastasize in pleura or pericardium, frequently with more metastatic sites than other molecular types, and predominant central nervous system (CNS) metastases [20-22].

### Identifying the main mechanisms of resistance to ALK TKI

**FIGURE 2:**
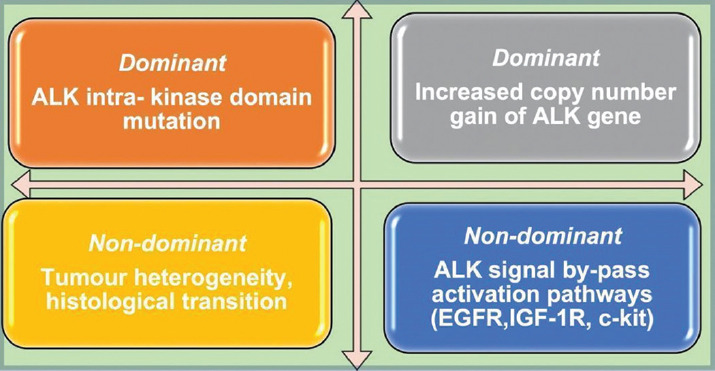
Describing the most common mechanisms of resistance to anaplastic lymphoma kinase tyrosine kinase inhibitors divided into dominant and non-dominant category.

Despite the major therapeutic improvement of ALK TKI in NSCLC, disease progression after initial benefit has been described due to the development of resistance mechanisms with clinically progressive disease and a variable range of aggressiveness [23-25]. The mechanisms of resistance can be divided into “*de novo*” or acquired depending on the timeline of occurrence and according to the involvement of ALK are classified into ALK-dependent or ALK-independent mechanisms [26,27]. Regarding the ALK dependency, ALK-dependent “on-target” tumors are driven by ALK signaling, while ALK “off-target” means that the driver mutation and the tumor cells are based on a different mechanism such as by-pass signaling pathways activation, drug efflux mechanisms, or histological transition (such as small cell transformation) [28,29]. Secondary resistance can be divided into dominant (ALK intra-kinase domain mutation, increased copy number gain of ALK gene) and non-dominant such as tumor heterogeneity, bypass signaling pathways activation such as the EGFR, Kirsten rat sarcoma viral oncogene homolog (KRAS), v-kit Hardy-Zuckerman 4 feline sarcoma viral oncogene homolog (KIT), met proto-oncogene (MET), and insulin-like growth factor 1 receptor (IGF-1R), as presented in Figure 2 [30,31]. Studies have shown that multiple mechanisms of resistance can occur simultaneously by the identification of epithelial-to-mesenchymal transition (EMT) in ALK kinase domain mutations [31,32]. Furthermore, the presence of tumor protein 53 (TP53) mutations in ALK-rearranged NSCLC defines a category with the instability of the chromosomes, conferring a prognostic role and determines pathogenic aberrations which can co-occur [32-34]. Clinical data suggests that the baseline level of TP53 is correlated with worse PFS, a shorter OS, and a more aggressive disease [35-37]. Furthermore, other genes involved with low frequency were identified, such as: BRAF, FGFR2, MET, NRAS, and PIK3CA [38,39]. Most of the secondary resistance mutations from ALK tyrosine-kinase domain currently are mainly point mutations such as the gatekeeper mutation L1196M, G1269A, and G1202R [40,41]. Although in NSCLC patients, personalized therapy was developed using not only using ALK gene but also EGFR as molecular target, the mechanisms of resistance between these two genes are completely distinct due to different tumor biology including genomic instability and different oncogenic dependency. Furthermore, the TKI implemented has contrasting properties determined by the binding modalities and inhibiting potency of the molecules [42,43]. Among the by-pass signaling mechanisms which confer resistance to ALK inhibition, the HER family activation is one the most important identified [43,44]. The identification of EGFR in post-crizotinib resistance, even in the absence of mutations, suggests the activation of this pathway by the paracrine stimuli, such as neuregulin 1 (NRG-1) [44,45].

Despite the development of TKI resistance mechanisms previously described, targeted therapy is considered the main therapeutic approach in ALK-rearranged NSCLC and literature data reports a median survival time of approximately 4 years in metastatic patients, significantly higher in comparison with chemotherapy [46,47].

### Exploring ALK TKI and their particularities

**FIGURE 3:**
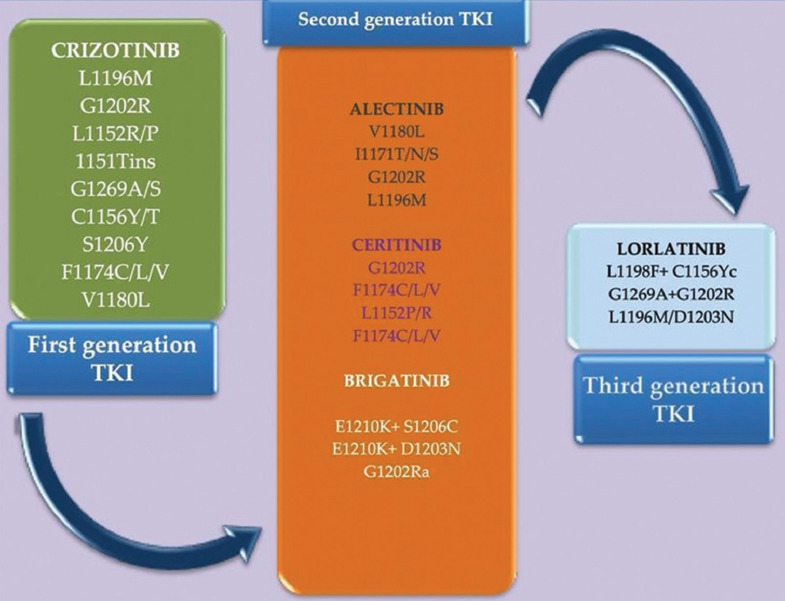
The most common anaplastic lymphoma kinase (ALK)- dependent resistance mutations associated with therapeutic resistance to ALK tyrosine kinase inhibitors (TKI). Each generation of ALK TKI is presented in the scheme with the approved drug and the main point mutations associated with resistance and different colors were used to highlight these point mutations.

The therapeutic implementation of the ALK TKI agents generates a clinical controversy based on the appropriate use, which should be guided by the potency and the CNS penetration, a frequent site of metastasis in patients with ALK rearrangement [48-51]. The main studies which have led to the approval of these molecules are summarized in Table 1. Another two molecules, ensartinib and entrectinib have been discovered and are currently into clinical trials, but currently, they have not gained FDA approval. Ensartinib is a potent ALK inhibitor with high CNS penetration and potential synergistic activity with the mTOR inhibitor rapamycin. Entrectinib is a new generation inhibitor of multiple targets, such as neurotrophic tyrosine kinase, receptor type 1, neurotrophic tyrosine kinase, receptor type 2, neurotrophic tyrosine kinase, receptor type 3, ROS proto-oncogene 1 (ROS1), and ALK which has been approved in NRK and ROS1 positive NSCLC [52-54]. Among the mechanisms of resistance, there are several point mutations identified for each molecule, which may be common in the same TKI generation or particularly, as it will be detailed in the following paragraphs and mentioned in Figure 3 [55-58]. Studies have shown that the development of ALK resistance mutations can be induced by the use of multiple sequential lines of TKI [58-60]. After exposure to crizotinib, ALK gene copy number gain has been observed, but it was not considered to have a clinical impact and therefore was not reported as resistance mechanism after more potent ALK TKI [61]. After developing resistance to second-generations ALK TKI, literature data have proven that half of the mutations are acquired, out of which G1202R is the most frequent [62,63]. The concept of progressive multistep genetic complexity was implemented due to the identification of two or more mutations in patients who benefited from ALK TKI molecules of the first- and second-generation [64].

**TABLE 1:**
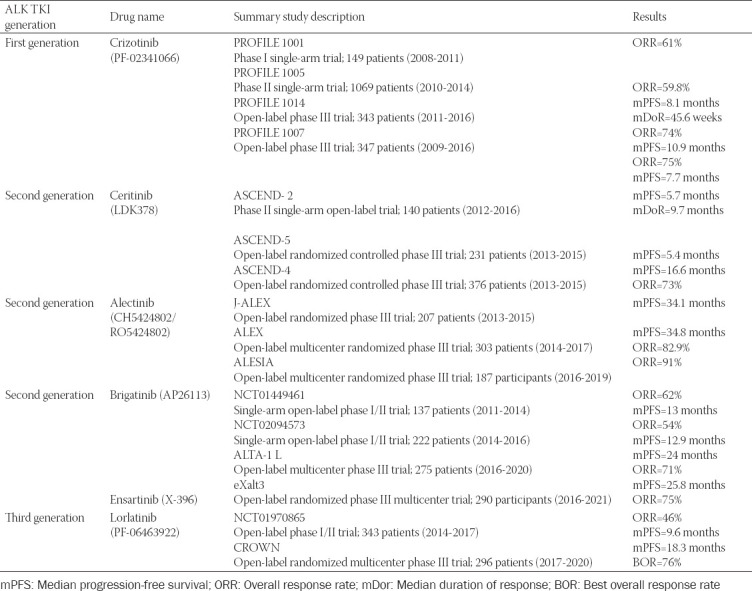
A summary description of the main studies and results of the ALK TKI in patients with ALK-positive NSCLC [70]

Crizotinib is an oral TKI inhibitor of the first generation TKI with recommended oral dose is of 250 mg twice daily in a 28-day cycle until disease progression or no longer tolerated by the patient and its metabolism involves the CYP3A 4/5 mechanisms, the most common side effects include: Hepatotoxicity, interstitial lung disease (pneumonitis), prolongation of QT interval, bradycardia, visual toxicity (including visual loss), and gastrointestinal affection, which require either dose reduction or permanent interruption [65]. Ceritinib is a second-generation ALK TKI with a therapeutic dose of 450 mg orally once daily with a mainly hepatic metabolism through the enzyme complex CYP3A. Apart from the common adverse effects mentioned for the first-generation TKI, subsequently to the treatment with ceritinib the following toxicities have been identified: Hyperglycemia, pancreatitis, and embryo-fetal toxicity [66]. Alectinib is recommended in a twice-daily dose of 600 mg and its particular side effects include renal impairment, severe myalgia, and creatine phosphokinase elevation [67]. Brigatinib is approved with a dose of 90 mg once daily for the first 7 days of the treatment, increasing afterward at 180 mg once daily. It presents the common side effects of the entire class but also hypertension [68]. Lorlatinib is a third-generation TKI that is metabolized mainly by CYP3A4 and UGT1A4 pathways recommended in a dose of 100 mg once daily. The adverse effects of this class are different from the previous described and include CNS side effects (such as seizures, hallucinations, and changes in cognitive function), hyperlipidemia, and atrioventricular block [69].

### Describing mechanisms of resistance in the first, second, and third generation of ALK TKI

Crizotinib was the first oral TKI molecule approved in 2011 by the FDA for metastatic NSCLC patients with ALK mutation, but almost a third of the patients had developed primary or secondary resistance within 1-2 years [71]. Crizotinib was first implemented to inhibit the c-MET pathway but has also proved important activity against ALK and ROS1 gene [72]. Furthermore, due to the promising results obtained from the clinical trials, crizotinib was approved in November 2013 with the indication of a second therapeutic option for patients with ALK mutation and progressive disease after platinum doublet treatment. About half of the patients developed metastasis in the CNS, considered the main or the exclusive site of disease progression because crizotinib has a decreased capacity to cross the blood-brain barrier [73]. One of the most frequent mutations identified in crizotinib resistance patients was L1196M gatekeeper mutation, analogous to EGFR T790M, which alters the gatekeeper residue at the bottom of the ATP-binding pocket and subsequently inhibits the binding with TKI. In addition, it has been discovered that patients who harbor L1196M mutation tend to have a shorter PFS [74]. The G1202R solvent front mutation has been identified in patients treated with first-generation ALK-TKI and determines the alteration of the binding activity of crizotinib [75,76]. Literature data have identified that the most common mutations in crizotinib-resistant patients are the following: L1152R, C1156Y, F1174C, L1196M, D1203N, C1156Y, and G1269A as well as EGFR activation working as the bypass pathway [77,78]. Other mechanisms of resistance to crizotinib have been identified: ALK gene amplification and copy number gain, new mutations such as 1151Tins and point mutation S1206Y in the solvent front of the kinase domain [79,80]. Intrinsic factors such as the concomitant KRAS (Kirsten rat sarcoma 2 viral oncogene homolog) mutations, MYC amplification, and the Bim deletion polymorphism could determine primary resistance to crizotinib [80,81]. In the acquired resistance, increased ErbB signaling through phosphorylation has been involved as well as the activation of the IGFR-1R pathway, EMT, and autophagy. The extended research in this domain has determined the identification of new mutation genes, such as CSMD3, CDKN2, MAGI1, CREBBP, DOT1L, PBX1, and PRKDC [82,83]. Potential mechanisms of resistance to crizotinib are represented by the activation of EGFR through the overexpression of the cKIT gene determined by its linkage to stem cell factor [83]. Activation of the PI3K/AKT/mTOR pathway has also been recognized as a resistance mechanism to crizotinib, possibly through increased autophagy of the ALK receptor. Synergy of crizotinib and a mTOR inhibitor in terms of inhibitory activity has been demonstrated in a cell line [84]. Therefore, due to the identification of crizotinib resistance, further ALK TKI molecules of the second and third generation have been implemented for the daily therapeutic approach [85].

Ceritinib was the initial ALK TKI molecule of the second-generation class approved to overcome resistance to crizotinib [86]. In 2014, ceritinib was indicated ALK-positive patients with disease progression on or intolerance to crizotinib and in 2017 was indicated as the first-line therapeutic setting. The mechanism of action is represented by the inhibition of the autophosphorylation of ALK gene and the molecular targets include IGF-1 R, InsR, and ROS1 and have an activity of 20 times higher against crizotinib–resistant tumor cell lines [87]. Ceritinib inhibits the most common ALK mutations, such as L1196 M, G1269A, I1171T, and S1206Y, which determine resistance to crizotinib [86,87]. In patients who progressed during ceritinib treatment, secondary mutations were detected such as G1202R, F1174 C/L, C1156Y, G1202del, and L1196M. The F1174L mutation which can be resistant to ceritinib but sensitive to alectinib and the I1171 mutation sensitive to ceritinib but resistant to alectinib has been discovered [88]. In addition, the F1174C/D1203N compound mutations found in patients treated with crizotinib and ceritinib have conferred resistance to these molecules and also to alectinib, brigatinib, and lorlatinib [89]. A combination between a MEK inhibitor and ALK TKI was developed due to ceritinib resistance identified by the MEK activating mutation (MAP2K1-K57N) [90,91].

Alectinib is a molecule from the second generation, which has proved antitumor effect on NSCLC patients with ALK rearrangement who have benefited previously from crizotinib which was approved in December 2015 and subsequently in November 2017 was indicated as first-line setting of patients with advanced ALK-positive NSCLC [92]. Due to its chemical structure, it has proved efficient for patients with crizotinib-resistant ALK mutations such as L1196M, F1174L C1156Y, G1269A, 1151Tins, and L1152R but not G1202R [91]. Potential mechanisms of resistance to alectinib include the activation of EGFR signaling pathway, increased activation of IGF1R, HER3 overexpression, and P2Y receptors (P2Y1 and P2Y2) which increase the levels of activated protein kinase C (PKC) [93]. At the relapse, among the acquired resistance mutations, G1202R, I1171T/S, and V1180L and L1196M were identified [94]. Another mechanism involved in the acquired ­resistance to alectinib was activation of the hepatocyte growth factor (HGF)/MET signaling pathway [95]. Recent studies have shown that *in vitro* inhibition of Src and MET restored the sensitivity to alectinib in patient-derived cell lines which have turned resistant to alectinib by the co-activation of these two mechanisms [96]. A potential resistance mechanism common for alectinib and crizotinib can be represented by the activation of HER3 pathway and overexpression of NRG1. [97]. Furthermore, human neuromedin U (NMU) gene is identified as a potential candidate which confers alectinib resistance in NSCLC patients, according to recent studies [98,99].

Brigatinib is a potent molecule from the second generation of ALK TKI with approval in 2017 for the indication of advanced NSCLC with ALK rearrangement with progressive disease on or intolerance to crizotinib. Furthermore, in 2020 it was approved as first-line treatment for ALK-positive metastatic NSCLC patients [100-102]. Brigatinib also inhibits ROS1 fusions and EGFR mutation (L858R) and has a remarkable activity on the CNS [102,103]. According to the clinical data, brigatinib is active against all the 17 known resistance mutations to the ALK gene, including G1202R and L1196M [104,105]. Among the mechanisms of resistance to brigatinib, gene mutations such as D1203, S1206, E1210K + S1206C, and E1210K + D1203N have been identified [106]. The molecular structure of brigatinib, which may lead to resistance development, is also influenced by the L1196M, G1269A, F1174L, and R1275Q mutations [107,108]. Furthermore, it has been discovered that the mutation G1202R occurs after previous exposure to ceritinib, alectinib, and brigatinib, meaning that lorlatinib is the only efficient therapeutic agent [108,109].

Ensartinib is a novel ALK TKI molecule of the second generation with improved action on CNS metastasis and is a potential option for the first-line setting according to the preclinical data [110]. Compared to the other ALK TKI, ensartinib is active against the following mutations: G1123S, L1198F, F1174, C1156Y, L1196M, S1206R, and T1151, but less potent against G1202R and G1269A [111]. Upon progressive disease, two mutations have been identified: E1210K and S1206F. Apart from the ALK inhibition, ensartinib is also active against MET, Axl, ABL, EPHA2, Leukocyte Receptor Tyrosine Kinase, ROS1, and STE20 like kinase [112].

Entrectinib is another second-generation ALK inhibitor candidate, which is able to penetrate the blood-brain barrier exhibiting, therefore, consistent intracranial activity under clinical research. In addition, apart from the ALK inhibition, entrectinib is also active against Tropomyosin receptor kinase A, Tropomyosin receptor kinase B, Tropomyosin receptor kinase C, and ROS1. The results of the clinical data are awaited to conclude the efficacy of this molecule in ALK TKI setting [113].

Lorlatinib is a molecule of the third-generation approved in 2018 by the FDA in the first-line setting for metastatic NSCLC patients and ALK rearrangement with progressive disease on crizotinib and other ALK inhibitors [114,115]. Due to its activity, it targets mutations, which determine resistance to the other ALK TKI, including G1202R solvent-form mutation, and facilitates CNS penetration, subsequently determining an increased survival rate [116]. After analyzing the post-progression samples, the literature data suggest that half of the patients developed compound mutations such as ALK-G1202R/L1196M, ALK-E1210K/D1203N/G1269A, and ALK-I1171N/L1198F [117]. Although it proves efficacy against C1156Y gene, resistant to crizotinib or ceritinib, a potential mechanism of resistance is conferred by the co-occurrence of L1198Fmutation [118,119]. More clinical data are awaited because the previous mentioned mutations narrow the therapeutic options; therefore, this molecule is under active research.

### The main methods of detecting ALK rearrangement and mechanisms of resistance in NSCLC

To provide the best therapeutic option, ALK gene rearrangement identification is essential. Several mechanisms of detection have been implemented, each with their advantages and limits, as described in Table 2. FISH testing with the ALK break-apart probe kit was the mandatory diagnosis test due to its use as a companion diagnostic tool in the clinical trials of crizotinib [120]. Although it has been considered the gold standard because it provides very good specificity, can be performed in small biopsy sample and it is used to validate and compare other ALK detection methods, FISH has disadvantages such as signal instability, expensiveness, difficulty in scoring, the long turnaround time, and the need for specific fluorescence microscopy and particular expertise to validate the results. Furthermore, FISH can only determine if there is a break in the ALK locus but does not have the ability to distinguish between the ALK fusion partners [121]. A modified FISH assay with filtration enrichment, filter-adapted FISH was implemented to identify ALK rearrangements on the CTC, but specific technological requirements are necessary for both CTC isolation and analysis, but due to the precise results obtained, it may become a non-invasive predictive biomarker [122].

**TABLE 2:**
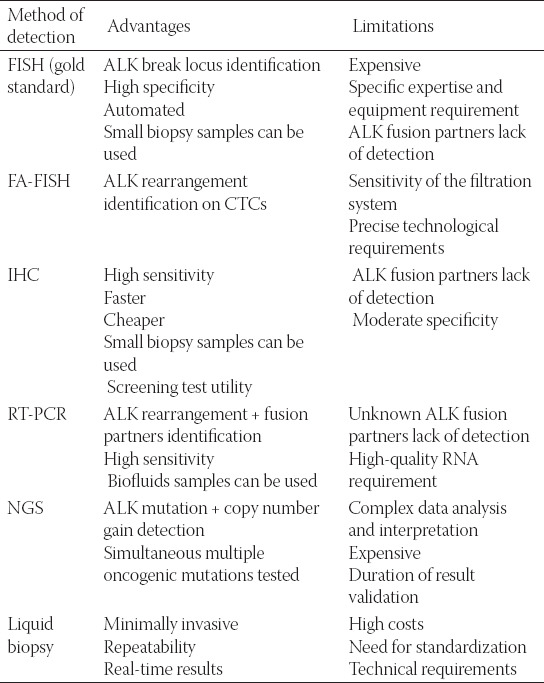
The main methods used to identify ALK mutation and mechanism of resistance described according to their main advantages and limitations

IHC has excellent sensitivity, with the advantage of being less expensive, following a simplified and rapid method than FISH with the utility as a screening test for diagnosis of NSCLC patients with ALK rearrangement, but it has poor sensitivity and it cannot identify the ALK fusion partner [123]. RT-PCR and NGS technologies allow the analysis of ALK gene rearrangement and its fusion partner. RT-PCR uses specific primers for identified ALK fusion partners, but a disadvantage is that several tests should be performed before identification of the ALK fusion partner variant and the unknown molecules could not be detected [124]. Furthermore, high-quality samples are needed which can determine either false-negative or positive results. RT-PCR was commonly used with screening purpose because of the high sensitivity and the applicability to biofluids, but it requires assay optimization for each new fusion partner and high-quality ribonucleic acid (RNA) [125]. NGS has the ability of identifying ALK mutations, copy number gain and provide identification of numerous oncogenic mutations at the same time in one assay with high sensitivity and reproducibility. At present, NGS has proved able to detect ALK fusion genes by sequencing the intron between exons 19 and 20, where they usually occur, but confirmation is required to gain approval for clinical application [126]. Tumor tissue samples used for the previous described testing methods are obtained from bronchoscopy or percutaneous lung biopsy and present several limitations, including the invasiveness acquisition, which may determine complications or may be insufficient and the tumor heterogeneity. To properly identify the mechanisms of resistance, liquid biopsy is considered of great significance due to its potential of detection against tumor heterogeneity and reflects the general particularities of the tumor. Its advantages include a less invasive procedure, which has a simple mechanism of interpretation providing real-time results and can be repeated at need [101,102]. CTC, circulating tumor DNA (ctDNA), and exosomes in body fluids are the main biomarkers which determine real-time resistance and can guide the follow-up treatment. The percentage of mutated CTCs, microRNAs, and proteins reflects the resistance mechanism developed by the activation of oncogenic drivers [124,125]. An important discovery was the detection of L1196M gene mutation on CTC at an early stage in patients who developed crizotinib secondary resistance [127]. Despite the low concentration in peripheral blood, CTC are ideal for detection, analysis, and overcoming tumor heterogeneity due to their distinct origin from the solid tumor or cancer sites. ctDNA is defined as tumor tissue-specific DNA fragment released in the bloodstream and its level is correlated with tumor progression, being the most frequent biomarker used in ALK-positive NSCLC resistance monitoring [123,124]. Studies have concluded that ctDNA NGS represents a non-invasive method of detecting targetable alterations and characterizing resistance mechanisms upon TKI progression [128]. Several detecting methods of TKI resistance are currently under research, such as exosome micro RNA because the concentration of exosomes in peripheral blood is greater than CTCs and it can also be identified in other body fluids besides serum and plasma [125,126]. In contrast, circulating ALK RNA analysis has proved low sensitivity due to the low blood stability [127]. A common method to detect resistance is the evaluation of tumor tissue before and after exposure to TKI by sequencing analysis. Furthermore, upon progression, an attempt to provide tissue sample or liquid biopsy is indicated [101,125]. On the other hand, studies reveal that plasma monitoring in ALK-rearranged NSCLC is feasible and could avoid re-biopsy from the tissue and monitoring plasmatic mutation levels could be used as a response parameter [128,129].

## DISCUSSION

The actual research in this domain explores the options of overcoming TKI resistance by providing future therapeutic directions. Preclinical studies have identified that inhibitors against HSP90 (heat shock protein 90), the molecular protein responsible for ALK fusion stability, such as 17-AAG, 17-DMAG, and ganetespib have antitumor efficacy. Furthermore, HSP90 inhibitors have proved superiority against wild-type EML4-ALK mutation, L1196M, and F1174L gene mutations [130]. Ganetespib, which is also active on ROS1 rearrangements and RET kinases, has been evaluated individually and in combination with crizotinib and other ALK TKI to overcome crizotinib resistance *in vitro* and *in vivo* [131]. Literature data suggest the use of ALK-TKI ceritinib and the EGFR-TKI afatinib for patients who have acquired ALK TKI resistance through EGFR pathway activation because afatinib restored the sensitivity of H3122-CER cells and subsequently increased apoptosis and the antitumor activity [124,132]. Studies have identified that crizotinib is active not only against ALK but also against MET, AXL, and MST1R (Macrophage Stimulating 1 Protein Receptor) genes and it may target other TKI which are potential drivers of resistance to ALK inhibition in H3122 cells [133]. Another therapeutic option under research is represented by metformin, an oral antidiabetic drug which reverses resistance to crizotinib through the inhibition of the IGF-1R signaling pathway [124,131]. At patients with crizotinib resistance, EMT is associated with decreased expression of miR-200c and increased expression of ZEB1 (Zinc Finger E-Box Binding Homeobox 1); therefore, it determines cross-resistance to new-generation ALK inhibitors alectinib, ceritinib, and lorlatinib. Furthermore, in patients with coexistence of resistance mutations and EMT, pretreatment with histone deacetylase (HDAC) inhibitor quisinostat has proved efficacy [126,134]. ALK and vascular endothelial growth factor receptor (VEGF-R) share reciprocal downstream signaling; therefore, simultaneous inhibition of ALK gene and VEGFR by the linkage between alectinib with afatinib can provide overcoming of ALK resistance [135]. Another combination therapy undergoing preclinical studies is between alectinib and bevacizumab in patients with NSCLC and ALK rearrangement, which have CNS involvement with at least targetable lesion, because bevacizumab reshapes tumor vasculature and subsequently adjusts the systemic and intracranial drug activity [127,135]. In recent studies, MYC proto-oncogene amplification has been associated with developing primary resistance to crizotinib; therefore, a combination of MYC-directed inhibition treatment such as CDK4/6 inhibitors may provide an alternative option. In NSCLC patients with ALK rearrangement and TP-53 mutation, MYC overexpression determined a potential MYC-dependent resistance mechanism [136,137]. For patients with crizotinib resistance due to the presence of C1156Y mutation, methionine residue (M-1199) may represent a targetable approach [138]. *In vitro* studies have developed a structural similarity of Alectinib (JH-VIII-157-02) which is active against many resistance mutations, including G1202R and has important CNS activity [139]. Another study concluded that activated HER family signaling and mediated EGFR activation by amphiregulin protein are mechanisms which determine resistance to ALK inhibitors, suggesting that the resistance mechanism is potentially reversible [124,138]. Another potential mechanism of resistance to ALK inhibition is represented by the P2Y purinergic receptors, which co-stimulate and activate the EGFR/MAPK signaling pathway and increase the activation signal through the PKC (protein kinase C) activation [20]. In addition, IGF-1R has a synergistic effect on ALK signaling due to its protein Insulin receptor substrate 1 (IRS-1) which inhibits the IGF1R/IRS1 pathway and increases the sensibility of the tumor cells to ALK targeting [29]. Preclinical data exhibit the synergistic effect of saracatinib (a dual Src and Bcr-Abl inhibitor) on ALK inhibition through its mechanism of phosphorylation which determines resistance when SRC kinase is upregulated. Actually, the mechanism of phosphorylation of SRC substrates was expanded after both first and second-generation TKI [139,140]. Regarding the molecular changes, a tumor suppressor gene NF2 (neurofibromin 2), which activates the bypass signaling of PI3K-AKT-mTOR pathway, was identified in mutant forms at the progression with crizotinib and determined a sensibilization at the inhibitory effect of mTOR, providing possible clinical implications [117]. Another option for overcoming ALK-TKI resistance is represented by the Yes-associated protein, a downstream effector of the Hippo pathway, due to its overexpression, which inhibits the therapeutic response to alectinib as suggested by the preclinical data [141,142].

Several phase I and II studies are currently studying the potential benefit and tolerability of the combination between ALK TKI and immune checkpoint inhibitors in lung cancer, such as ceritinib with nivolumab, alectinib with cobimetinib, lorlatinib with crizotinib and binimetinib, ceritinib with trametinib, alectinib with cobimetinib, and brigatinib with binimetinib and the results are highly awaited [47,143]. Research data have revealed that the expression of PD-L1 is 5 times higher in patients with ALK gene rearrangement; therefore, promising results could highlight the efficacy of anti-PD-1/PD-L1 antibodies in this category of patients. Silibinin treatment inhibited the upregulation of the programmed death-ligand 1(PD-L1) and EMT regulators in crizotinib-resistance cells, suggesting a potential improvement of ALK TKI resistant NSCLC patients with silibinin-based drugs [145]. Furthermore, combination therapies such as inhibitors of ALK and MAPK signaling pathways, ceritinib + CDK4/6 inhibitor, ceritinib + mTOR inhibitor, and alectinib + anti-angiogenesis inhibitor have potential mechanisms of overcoming resistance [47,143-146].

## CONCLUSION

We consider that identifying and overcoming mechanisms of resistance to ALK-rearranged TKI represents a promising research domain, which still requires continuous efforts to discover the remaining questions. In our opinion, this topic represents one of the most challenging of oncological research with unmet clinical needs so far.
